# The post hoc measurement as a safe and reliable method to age and size plethodontid salamanders

**DOI:** 10.1002/ece3.6748

**Published:** 2020-09-09

**Authors:** Enrico Lunghi, Simone Giachello, Raoul Manenti, Yahui Zhao, Claudia Corti, Gentile Francesco Ficetola, Joseph Gavin Bradley

**Affiliations:** ^1^ Key Laboratory of the Zoological Systematics and Evolution Institute of Zoology Chinese Academy of Sciences Beijing China; ^2^ Museo di Storia Naturale dell'Università degli Studi di Firenze Museo “La Specola” Firenze Italy; ^3^ Dipartimento di Scienze e politiche ambientali Università degli Studi di Milano Milano Italy; ^4^ Laboratoire d'Écologie Alpine (LECA) CNRS University Grenoble Alpes Grenoble France; ^5^ Biology Department Elizabethtown Community and Technical College Elizabethtown KY USA

**Keywords:** Amphibia, Caudata, morphometry, noninvasive, photography, snout–vent length, Urodela

## Abstract

The worldwide biodiversity crisis with the resulting need to increase species protection has led researchers to pursue and select survey methods that guarantee the best quality of data and produce the least negative effects on wild animals. Plethodontids are the most diverse family of salamanders; all species are very sensitive to human handling and noninvasive, but accurate, measurement methods are needed to reduce researchers’ impact. Here, we tested the reliability of a noninvasive post hoc method in estimating the snout–vent length (SVL) from photographs showing salamanders’ dorsal view. The correlation between the estimated snout–vent length (SVL_e_) and the conventional SVL was high (*R* ^2^ *_m_* = .81), and no significant difference occurred between operators with different experience. Finally, we list the numerous advantages for the use of SVL_e_ in terms of data quality and in reducing the stress caused to wild animals.

## Graphical abstract

We present an alternative methodology to age and size plethodontid salamanders from pictures.

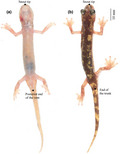

## 1 INTRODUCTION

Earth is experiencing the sixth mass extinction (Barnosky et al., 2011), an event widely exacerbated by human actions (Ceballos et al., 2015). Amphibians are among the most affected species as they often show the combined effects of high sensitivity to environmental alteration and low dispersal ability (Beebee & Griffiths, 2005; Catenazzi, 2015). Pathogenic fungi of the genus *Batrachochytrium* (*B. dendrobatidis* and *B. salamandrivorans*) are a particularly serious threat to amphibians, as they can wipe out entire populations (Garner et al., 2006; Martel et al., 2014). Many countries are now adopting rigorous policies to protect biodiversity (European Community, 1992), and researchers are developing survey techniques to minimize their impact on study species (Ficetola, Barzaghi, et al., 2018; Ficetola, Manenti, & Taberlet, 2019; Sharifi, Naderi, & Hashemi, 2013).

Handling wild animals does not only facilitate transfer of pathogens (Garner et al., 2006; Martel et al., 2014) but induces stress, provoking a cascade of negative effects on their immune system (Caipang, Fatira, Lazado, & Pavlidis, 2014), microbiome (Allen‐Blevins, You, Hinde, & Sela, 2017), and behavior (Bliley & Woodley, 2012). Amphibians are particularly susceptible to handling, and even short manipulations using gloves may strongly alter their internal temperature (Lunghi et al., 2016).

The most common method used to measure salamanders is snout–vent length (SVL), a method that requires handling individuals. This measurement is taken ventrally from the snout tip to the posterior opening of the cloaca (Bingham, Papenfuss, Lindstrand, & Wake, 2018). SVL aids in distinguishing life stage and size in salamanders; thus, SVL can be used to differentiate adults from juveniles by using the smallest sexually mature adult as a reference (Lanza, Pastorelli, Laghi, & Cimmaruta, 2006). This method is particularly useful for differentiating between life stages of plethodontid salamanders with direct development because adults and juveniles exhibit few other morphological differences (Wells, 2007). Generally, accurately measuring individuals in the field is challenging and aiming for high measurement precision usually leads to a high error rate because individuals continuously squirm when handled (Luiselli, 2005; Setser, 2007; Zweig, Mazzotti, Rice, Brandt, & Abercrombie, 2004). Terrestrial plethodontids require high humidity and cool temperatures to respire efficiently (Ficetola, Lunghi, et al., 2018; Spotila, 1972), and because plethodontids are lungless, respiration is mainly conducted through the skin (Wells, 2007); thus, prolonged handling likely reduces the amount of gas exchange. Additionally, each survey can last only for a defined time and there are limited chances to spot errors accrued during data collection. This increases the risk of obtaining inaccurate or even useless data (Brown, Kaiser, & Allison, 2018; Lunghi, Romeo, et al., 2019; Margenau, Crayton, Rucker, Jacobsen, & Brown, 2018). Here, we assess the reliability of a post hoc method based on photography of salamanders from the dorsal view that could be used as a proxy of SVL while avoiding aforementioned problems. Although similar approaches have been tested on different amphibian and reptile species (Bray & Allain, 2019; Drakeley, Lapiedra, & Kolbe, 2015; Greer & Wadsworth, 2003; Lowe & McPeek, 2012; Margenau et al., 2018), a test on the endangered European plethodontid salamanders of the genus *Hydromantes* (Wake, 2013) is missing. We also provide an estimation of the errors occurring among and within individuals’ measurements.

## 2 MATERIALS AND METHODS

The European *Hydromantes* are comprised of eight threatened species distributed in Italy and France (Lanza et al., 2006). These salamanders are strictly protected by national and international laws (European Community, 1992; Rondinini, Battistoni, Peronace, & Teofili, 2013) because multiple threats, such as habitat degradation, climate change, spread of pathogens, and poaching, are negatively impacting their populations (Lunghi, Corti, Manenti, & Ficetola, 2019; Mammola et al., 2019; Martel et al., 2014); therefore, specific authorizations are needed to conduct studies on these species. European *Hydromantes*, like most other plethodontids, are surface‐dwelling species able to maintain stable populations in subterranean habitats where they can find a suitable and stable microclimate (Camp & Jensen, 2007; Lunghi, Manenti, & Ficetola, 2015) as well as a safe place to reproduce (Bradley & Eason, 2019; Lunghi et al., 2018). However, the intrinsic features of subterranean environments (e.g., narrow passages, air moisture near saturation) represent a natural challenge for researchers (MacNeil & Brcic, 2017), making it very difficult to collect data on subterranean populations. Using noninvasive methods, such as photographing individuals for measurements and identification, may not only limit negative effects incurred by wildlife, but may also alleviate the complexities of prolonged surveys performed in “nonhuman friendly” conditions that characterize subterranean and forest environments where plethodontids are found.

**FIGURE 1:**
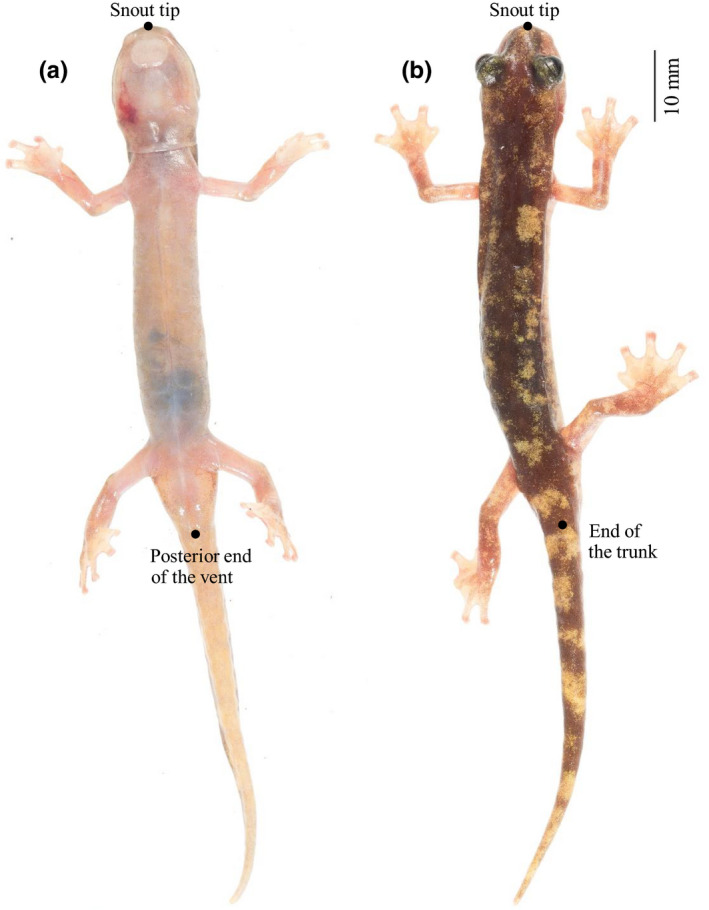
*Hydromantes flavus* (individual 1074777; Lunghi et al., 2020). (a) Ventral view, the points to measure SVL are marked. (b) Dorsal view, the points to measure SVL_e_ are marked

**FIGURE 2:**
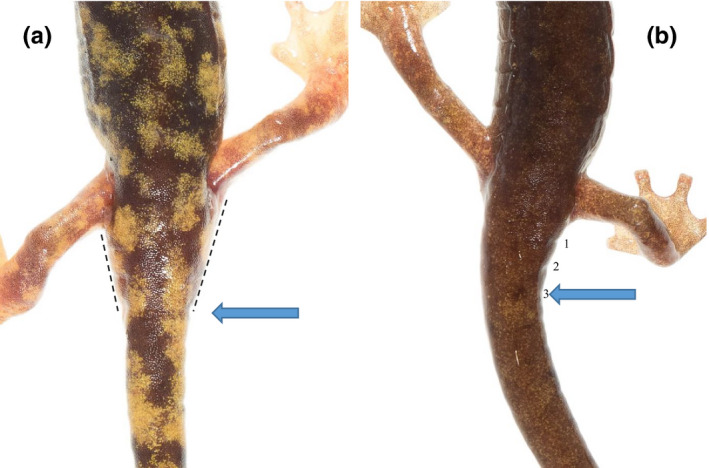
Diagnostic characters indicating where the trunk of *Hydromantes* ends. (a) The conical end of the salamander's body indicates the base of the tail; a natural “line” helps in identifying this point (*H. flavus*, individual 1074390). (b) In some cases, a few folds are visible behind the hind limbs; the posterior end of the cloaca roughly corresponds to the third fold (*H. genei*, individual 1033580)

In a previous study, Lunghi et al. (2020) produced a photographic database of European *Hydromantes*, providing high‐quality pictures of the dorsal pattern for more than 1,000 individuals of all eight species. The authors photographed salamanders in situ placing them in a white soft box to obtain high‐quality photograph (Lunghi et al., 2020). During this study, 22 individuals (~3 per species) were randomly selected and photographed ventrally (Figure 1a). Before photographing the salamanders, Lunghi et al. (2020) held them for 30 s in hand to increase their body temperature, which caused a short thermal shock that made them calm for a few seconds (Lunghi et al., 2016). These digital images were used to measure the 22 salamanders using the program ImageJ (Figure 1a). Dorsal and ventral images of each individual were paired. We measured SVL of each individual using images from the ventral view and used it as reference of a “true” measure (Margenau et al., 2018). Then, we asked 31 volunteers with different backgrounds (students or experienced herpetologists) to use ImageJ and estimate the SVL_e_ from the dorsal view (Figure 1b). Measures of SVL and SVL_e_ were taken to the nearest mm. All participants were given a manual that explained the measuring process, particularly how to recognize the diagnostic characters indicating the position of *Hydromantes*’ cloaca from the dorsal view, herein described. Located ventrally in the area between the hind legs and the tail is the cloaca (Figure 1a), which is the landmark used to measure SVL. Dorsally, this area has a conical shape that narrows from the hind legs to the tail base (Figure 2a). In some individuals, this area shows lateral “folds,” of which the third from the hind limb roughly corresponds to the tail base (Figure 2b), and thus the posterior end of the cloaca used in SVL. Photographs were provided without any additional information to ensure unbiased measurements (MacCoun & Perlmutter, 2015).

We used linear mixed models (LMMs) (function *lme* of the R package nlme; Pinheiro, Bates, DebRoy, Sarkar, & Team, 2016; R Development Core Team, 2019) to evaluate the relationship between SVL and SVL_e_ in European *Hydromantes*. The salamanders’ SVL_e_ was used as the dependent variable and the SVL as the independent variable. The experience of operators (yes/no), together with the identity of operators, salamanders, and species, were assigned as random factors. The correlation coefficient between SVL_e_ and SVL was calculated with the function *r.squaredGLMM* of the R package MuMIn (Bartoń, 2016). We calculated the mean squared error (*MSE*) between SVL and SVL_e_ and assessed whether it significantly differed from zero. We used LMM to evaluate the relationship between the *MSE* (dependent factor) and SVL (independent factor); considering that the operator experience may affect measurement equality between SVL and SVL_e_, we added “Expert” as a further independent variable to assess whether there were differences in SVL_e_ between students and experienced herpetologists. Random factors were the identity of operators and salamanders, and the species. Likelihood of fitted LMM objects was assessed using the function *anova*.

## 3 RESULTS

**FIGURE 3:**
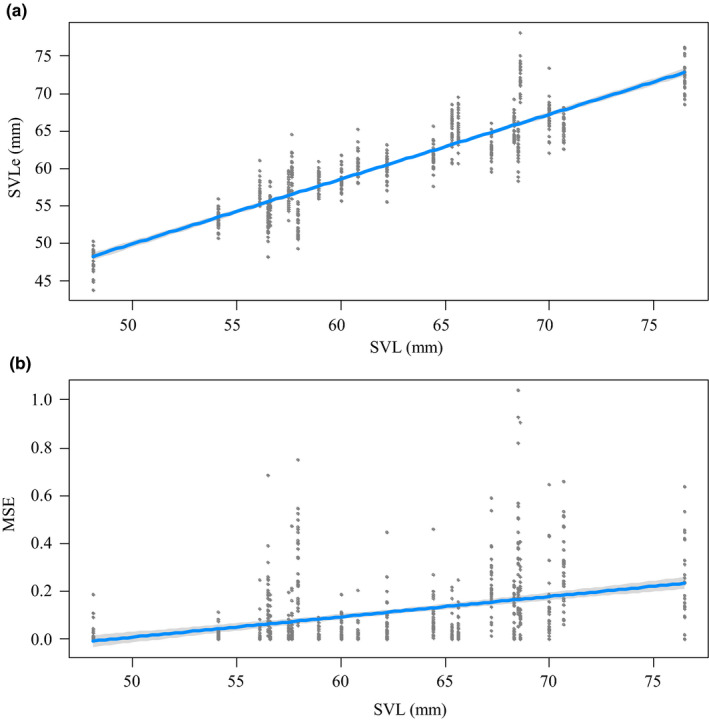
Correlation plots of SVL against (a) SVL_e_, and (b) against MSE, created with R package visreg (Breheny & Burchett, 2017)

The correlation between salamander SVL_e_ and SVL was high (*F*_1, 464_ = 3,154.56, *p* < .001; *R* ^2^_m_ = .81) with a regression slope of 0.87 (95% CI 0.83–0.90) (Figure 3a). The *MSE* was significantly different from zero (one‐way *t* test, *t* = 19.719, *df* = 681, *p* < .001; 95% CI 0.102–0.124) and the SVL_e_ was on average 2.69 mm (±0.08 *SE*) smaller than SVL. We found a weak, although significant correlation between *MSE* and salamanders’ SVL (*F*_1, 464_ = 115.78, *p* < .001; *R* ^2^_m_ = 0.14) with a regression slope of 0.008 (95% CI 0.007–0.01), while no effect of operator experience was detected (*F*_1, 29_ = 1.83, *p* = .186); the *MSE* was slightly higher in large salamanders (Figure 3b). For each salamander, there was an overall deviation from the average SVL_e_ of only 1.6 mm (±0.03), corresponding to 2.7% of the average salamanders’ SVL_e_.

## 4 DISCUSSION

Besides the reliable estimation of the “true” SVL, the use of SVL_e_ shows some noteworthy advantages. For example, the time available to measure SVL_e_ from pictures is virtually endless, whereas data collection in the field is more time restrictive. As mentioned above, measuring SVL in the field is difficult and may be prone to increased error rate (Guo, Chen, Zhang, Pan, & Wu, 2016; Luiselli, 2005; Setser, 2007). Once a salamander has been released, SVL cannot be measured again, so unnoticed errors that occurred while measuring will become part of the dataset (Brown et al., 2018). Furthermore, prolonged handling stress produces negative effects on animal health (Allen‐Blevins et al., 2017; Bliley & Woodley, 2012; Caipang et al., 2014; Lunghi et al., 2016). Therefore, photographing salamanders and the post hoc measurement of SVL_e_ facilitates easier measurements, requires shorter handling time with images of sufficient quality (Bradley, 2018; Lunghi et al., 2020), and is a method available to a wide number of operators (Margenau et al., 2018; Miyazaki et al., 2014). Nonetheless, such approach is particularly suitable for aquatic species, as operator can easily take measurements of individuals without removing them from water (Gutierrez, Guess, & Pierce, 2018).

The repeated SVL_e_ measurements performed on each salamander provided information on the robustness of this method. An error of 1.6 mm occurring when multiple operators measure SVL_e_ of each salamander (corresponding to 2.7% of the salamander “true” SVL) is likely lower than what can be obtained from a direct measurement of SVL for plethodontids in the field. Field measures of SVL (with no anesthetics) for snakes resulted in an average error of 2.5% (Setser, 2007). Error among operators in measuring SVL was about 1.5% for alligators (Zweig et al., 2004). In both cases, the size of measured animals was 8–30 times larger than our salamanders (on average ~ 62 mm); this highlights the ability of this approach to provide highly precise measurements for smaller animals. We observed that the *MSE* slightly increases with salamander size (Figure 3b). It may be possible that measurement inequality exists between SVL and SVL_e_, and such error proportionally increases with size (Hayek & Heyer, 2005).

Besides the multiple advantages of the proposed approach, a few general challenges should be noted. The main challenge is to obtain a picture of suitable quality to allow for the post hoc analysis. Artificial lighting is fundamental to highlight body details, and poor illumination can produce useless images (Lunghi, Romeo, et al., 2019). Photographs shot perpendicular to the longitudinal axis of the salamander increases the precision of measurements. *Hydromantes* are usually found under logs and stones or over complex surfaces (i.e., cave walls), and thus, their placement in a standardized area (i.e., flat surface allowing a perpendicular photograph) was crucial to completely flatten the animal and photograph from the proper angle (Lunghi et al., 2020). Nevertheless, some errors may also occur when measuring individuals from pictures. Indeed, particular attention should be paid during the setting of picture scale and when choosing start/end points, as multiple potential errors can accrue during this process.

## 5 CONCLUSIONS

We provided evidence of the reliability of the estimation of SVL from the dorsal view in *Hydromantes* salamanders in a noninvasive manner. We also reported all positive aspects that justify the best trade‐off between quality of data and disturbance caused to wild animals. Besides the reliability of measurements obtained with this approach, problems inherent to prolonged field activities may be alleviated and researchers can verify all measurements to spot any potential error.

## CONFLICT OF INTERESTS

None declared.

## AUTHOR CONTRIBUTION

**Enrico Lunghi:** Conceptualization (lead); Data curation (lead); Formal analysis (lead); Investigation (lead); Methodology (lead); Project administration (lead); Visualization (lead); Writing‐original draft (lead); Writing‐review & editing (lead). **Simone Giachello:** Investigation (supporting); Writing‐review & editing (supporting). **Raoul Manenti:** Investigation (supporting); Writing‐review & editing (supporting). **Yahui Zhao:** Investigation (supporting); Writing‐review & editing (supporting). **Claudia Corti:** Investigation (supporting); Writing‐review & editing (supporting). **Gentile Francesco Ficetola:** Investigation (supporting); Writing‐review & editing (supporting). **Joseph Gavin Bradley:** Conceptualization (supporting); Investigation (supporting); Writing‐review & editing (supporting).

## Supporting information

Appendix S1

## Data Availability

Data used in this paper are provided as Appendix S1. To retrieve the original code of individuals: https://figshare.com/s/066225d0977ed93c1f6b.
